# A Natural Fiber Complex Reduces Body Weight in the Overweight and Obese: A Double-Blind, Randomized, Placebo-Controlled Study

**DOI:** 10.1002/oby.20244

**Published:** 2012-07-26

**Authors:** Barbara Grube, Pee-Win Chong, Kai-Zhia Lau, Hans-Dieter Orzechowski

**Affiliations:** 1 Practice for General MedicineBerlin, Germany; 2 Research & Development Department, InQpharm Europe Ltd.London, UK; 3 Institute of Clinical Pharmacology & Toxicology, Charité UniversitaetsmedizinBerlin, Germany

## Abstract

**Objective:**

A proprietary natural fiber complex (Litramine IQP G-002AS) derived from *Opuntia ficus-indica*, and standardized on lipophilic activity, was previously shown in preclinical and human studies to reduce dietary fat absorption through gastrointestinal (GI) fat binding. Here, we investigated the efficacy and safety of IQP G-002AS in body weight reduction.

**Design and Methods:**

One hundred twenty-five overweight and obese adults participated in the study. Subjects were advised on physical activity, and received nutritional counseling, including hypocaloric diet plans (30% energy from fat and 500 kcal deficit/day). After a 2-week placebo run-in phase, subjects were randomized to receive either 3 g/day of IQP G-002AS (IQ) or a placebo. The primary endpoint was change in body weight from baseline; secondary endpoints included additional obesity measures and safety parameters.

**Results:**

One hundred twenty-three subjects completed the 12-week treatment phase (intention-to-treat (ITT) population: 30 male and 93 female; mean BMI: 29.6 ± 2.8 kg/m^2^ and age: 45.4 ± 11.3 years). The mean body weight change from baseline was 3.8 ± 1.8 kg in IQ vs. 1.4 ± 2.6 kg in placebo (*P* < 0.001). More IQ subjects lost at least 5% of their initial body weight compared to placebo (*P* = 0.027). Compared with placebo, IQ also showed significantly greater reduction in BMI, body fat composition, and waist circumference. IQ was well tolerated with no adverse reactions reported.

**Conclusions:**

These results suggest that the natural fiber complex Litramine IQP G-002AS is effective in promoting weight loss.

## Introduction

Obesity, the fifth leading risk of death worldwide is defined as an excessive fat accumulation that is associated with chronic medical conditions, which reduces life expectancy (1). It results from a level of energy intake, which exceeds the body's energy expenditure. Obesity and overweight are attributed to 44% of diabetes cases, to 23% of ischemic heart disease cases, and to 7-41% of certain cancers, such as colon cancer and breast cancer (1). Obesity is considered as an epidemic public health problem irrespective of genders, ethnicity, and age, affecting one in 10 adults worldwide (2).

There are currently 100 million overweight and obese adults in the United States, while the Multinational Monitoring of Trends and Determinants in Cardiovascular Disease (MONICA) project suggested that at least 15% of men and 22% of women in Europe are obese. Similar data are reported from many developing countries (including China, Malaysia, and part of South America). Unlike the developed countries, the socioeconomic status in these developing countries is positively correlated with the prevalence of obesity, which is regarded as an indicator of wealth (3).

The tremendous increase of obesity prevalence and incidence in the past two decades has cast a shadow over the world's socioeconomic state (4). Obesity is associated with depression (5-,6,7), which may be caused by disparagement of body image and negative emotional reactions to dieting (8). According to a 2010 review, total direct and indirect annual costs of obesity in the United States was at least USD 215 billion (9); while the total direct and indirect annual costs of obesity in 15 European Commission countries was ∼32.8 billion Euros in 2002 (10).

However, reducing the total caloric intake can prevent obesity and overweight. Previous studies have suggested that, apart from high carbohydrate intake, excessive intake of dietary fat (along with insufficient physical activity) plays a major role in causing obesity (11). Therefore, treatments to reduce the intestinal uptake of dietary fat continue to be an effective approach in weight management.

However, there is limited choice of effective and safe agents for reducing dietary fat absorption. Lipase inhibitors are generally approved by the health authorities in promoting weight loss by reducing the absorption of dietary fat via pancreatic lipase inhibition (12-15). However, this pharmacotherapy is associated with gastrointestinal (GI) side effects such as fecal incontinence, flatus with discharge, oily spotting (13,16-17), and liver injury (18). Meanwhile, nonpharmacological options, such as intake of dietary fiber, are of growing interest. However, poor product characterization and lack of evidence from clinical research have questioned efficacy of dietary fiber.

Litramine IQP G-002AS is a natural fiber complex derived from *Opuntia ficus-indica*, enriched with additional soluble fiber from *Acacia spp*. IQP G-002AS is standardized for its lipophilic activity and has been shown to reduce the dietary fat absorption through GI fat binding. The lipophilic IQP G-002AS binds to dietary fat, forming fat−fiber complexes, which are not absorbed by the intestine and, hence, are eliminated (19).

The fat-binding efficacy of IQP G-002AS was previously shown in *in vitro* GI models, animal studies, and human studies, where IQP G-002AS showed a reduction of dietary fat absorption up to 27% (19).

In this study, we investigated the efficacy and safety of IQP G-002AS in a randomized controlled trial to test the hypothesis that the intake of IQP G-002AS promotes increased weight loss compared to placebo in overweight and moderately obese human subjects.

## Methods and Procedures

### Subjects

Subjects were recruited through local press advertisements. Eligible subjects included obese and overweight (25 ≤ BMI ≤ 35) males and females, between 18 and 60 years old. Women of childbearing age were included with their agreement to use appropriate birth control measures during the entire study duration. Subjects with known sensitivity to the ingredients of the study medication were excluded from the study. Other exclusion criteria included presence of any GI disease, history of eating disorder, intake of antiobesity drugs, use of medication that affects GI function, alcohol abuse, smoking cessation in the previous 6 months, history of cardiac diseases, history of renal diseases, participation in similar studies or weight loss programs within the 6 months before this study, and pregnant and lactating women.

All subjects gave written informed consent voluntarily. The clinical investigation was approved by the ethics committee of the Charité–Universitätsmedizin Berlin and was performed in compliance with EN ISO 14155, the Declaration of Helsinki (Somerset, 1996) of the World Medical Association and the Guideline for Good Clinical Practice (CPMP/ICH/135/95).

### Study intervention

This double-blind, randomized, placebo-controlled, clinical investigation was conducted at two study centers in Germany from August 2010 to December 2010. Germany is one of the European countries with a significant proportion of overweight and obese subjects; 61.7% of German males and 45.2% of German females have a BMI of ≥25 kg/m^2^ (20).

The study comprised of a 2-week placebo run-in phase and a 12-week treatment phase. The run-in phase was meant to assess the compliance of the study subjects to the treatment (based on cumulative placebo tablets consumption) and dietary regime. Upon completion of the 2-week run-in phase, subjects who had a treatment compliance of at least 80%, experienced body weight reduction and who had less than 20% deviation from the prescribed daily calorie intake were randomized in a 1:1 ratio into either the IQP G-002AS group (IQ) or the placebo group. Randomization was done using a block size of 4 by an independent biostatistician using the randomization scheme BIAS for Windows V9.2 (http://www.bias-online.de).

During the 12-week treatment period, subjects received either two tablets of 500 mg of IQP G-002AS or a matching placebo, three times a day. Subjects were instructed to consume the study medication after breakfast, lunch, and dinner. The placebo tablet, identical in appearance to IQP G-002AS, contained 500 mg of microcrystalline cellulose as the replacement for the active ingredients. They were packed in bottles and labeled by an independent pharmacist for each subject according to the randomization schedule. All subjects and study personnel were not informed of the study group assignment.

All subjects were instructed to maintain a nutritionally balanced and mildly hypocaloric diet throughout the 2-week run-in phase and the 12-week treatment phase. The mildly hypocaloric diet plan was developed by the German Heart Institute, Berlin, Germany. It provided 30% of energy from fat, 55% of energy from carbohydrate, and 15% of energy from protein. The daily energy requirement was evaluated (for each subject) according to the equation of the Institute of Medicine (IOM) of The National Academies, based on age, gender, body weight, and physical activity rate (21). The calories contained in the mildly hypocaloric diet were equivalent to the estimated daily energy requirement, minus 500 kcal. In addition, subjects were advised to gradually increase their physical activity (30 min of moderate intensity physical activity such as walking or cycling).

Diet plans were distributed to all subjects at baseline and instructions were given at 4-week intervals, throughout the 12-week treatment period. Subject diaries were issued at baseline, week 4, and week 8, and subject diaries were evaluated at every visit, except baseline.

### Screening

The screening visit included detailed medical history, general physical examination, and measurements of blood pressure, heart rate, body weight, height, waist circumference, and body fat content. Blood was taken for clinical chemistry, hematology, lipid profile, and pregnancy test. All blood samples were analyzed centrally (Medizinisch-Diagnostische Institute, Berlin, Germany).

### Efficacy parameters

The primary efficacy parameter was change in body weight (kg) at 12 weeks compared to baseline. Body weight was measured using a calibrated weighing scale (Tanita BC-420 SMA; Tanita, Tokyo, Japan) in subjects wearing underwear and no shoes, at baseline and at 4-week intervals, throughout the 12-week treatment period.

Secondary efficacy parameters included proportion of subjects lost at least 5 and 10% of baseline body weight; reduction in mean BMI; reduction in mean waist circumference, which was measured at the level midway between the lateral lower rib margin and the iliac crest; change in body fat content (kg and %), which was measured by bioimpedance method using a validated electronic weighing scale Tanita BC-420 SMA. These parameters were also measured at baseline and at 4-week intervals, throughout the 12-week treatment period.

### Safety parameters

Safety assessments consisted of vital sign measurements (such as heart rate and resting blood pressure) and blood parameter evaluation including: clinical chemistry (electrolytes, fat-soluble vitamins, liver and kidney function, and purine metabolism), hematology and lipid profile (total cholesterol, triglycerides, low-density lipoprotein, and high-density lipoprotein). Heart rate and resting blood pressure were evaluated at each visit using a standard device; while the blood parameters were evaluated twice, at screening and upon the completion of 12-week study. The subjects also evaluated the tolerability of study medication subjectively, at the end of the study. All adverse events were recorded, regardless of their causality to treatment.

### Statistical analyses

The efficacy evaluation of IQ was based on the null hypothesis that there was no difference between IQ and placebo in mean reduction in body weight.

The sample size was calculated based on the primary endpoint, change in body weight from baseline. The minimum sample size of 125 subjects (with adjustment of 30% drop-out) provided 80% power of study to detect a weight loss difference of 1.78 kg between groups, at a significance level of 0.045. As pilot trial has not been performed with IQ, the sample size estimation was based on limited published literature (22-,23). Therefore, an unblinded interim analysis was considered necessary. Based on the method of Wittes-Brittain, the significance level was adjusted to 4.5% (two-tailed test), which fulfilled the requirement of the O'Brien-Fleming nominal significance level. The interim analysis (performed after half of the subjects had completed the 12-week study) indicated that sample size adjustment was not required.

Demographic and baseline characteristics of the efficacy and safety variables were first assessed based on the descriptive statistics. All primary and secondary variables as well as the safety variables were evaluated as relative change within each group. Parametric analysis, such as independent *t*-test was used primarily to determine the difference between groups. Repeated measures ANOVA was used to evaluate all efficacy variables in a repeated measurements design. The difference between groups of categorical variables was performed by the *χ*^2^ statistics. In addition, subjects were categorized into overweight (BMI <30 kg/m^2^) and obese group (BMI ≥30 kg/m^2^) and subgroup analysis was performed to determine the efficacy of IQP G-002AS on obese and overweight subjects only, discretely.

For all statistical analyses, *P* < 0.05 level of significance (two-sided) was assumed. All values are presented as mean ± s.d., unless otherwise indicated. Efficacy and safety variables were analyzed in the intention-to-treat (ITT) population, which is defined as subjects who had completed at least one efficacy assessment. All data were analyzed using the SPSS Statistic software, version 19.0 (SPSS, Chicago, IL).

## Results

**FIGURE 1:**
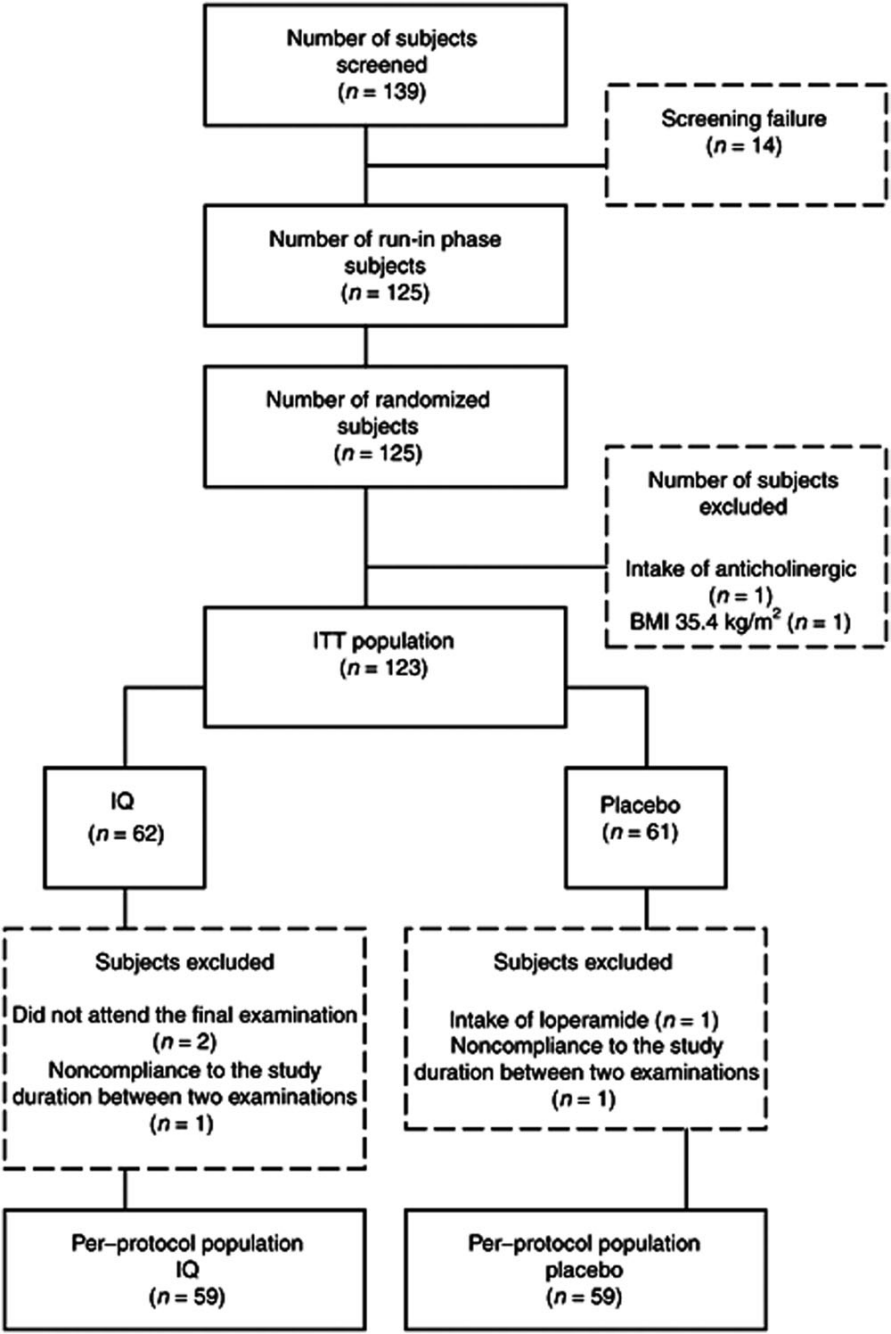
Study design and disposition of subjects. ITT, intention-to-treat.

One hundred thirty-nine subjects were invited for screening, out of those 125 were recruited. All subjects recruited showed compliance in the run-in phase and were randomized. The ITT population consisted of 123 subjects: 62 IQ subjects and 61 placebo subjects. One subject was excluded due to the intake of anticholinergic medication and the other because of BMI of 35.4 kg/m^2^. The demographic characteristics of the ITT population are shown in Table 1. The baseline characteristics of IQ and placebo were similar; there was no significant difference between groups in gender distribution, age, body height, body weight, and BMI. In the per-protocol analysis, 5 subjects were further excluded with 59 subjects each in IQ and placebo arms, due to reasons as detailed in Figure 1.

### Efficacy

**FIGURE 2:**
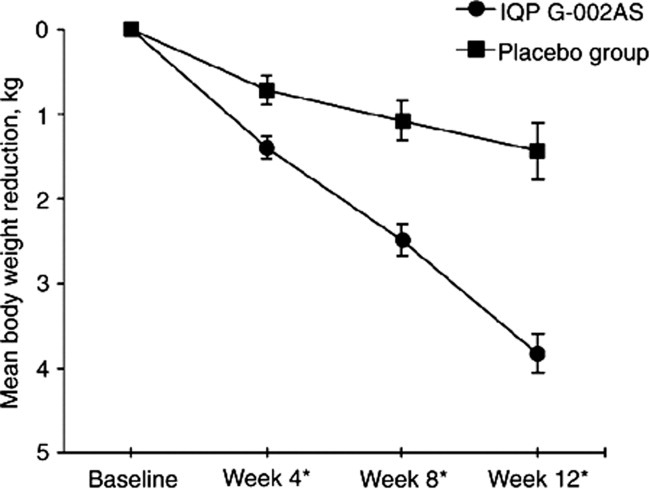
Body weight reduction over time. All data are presented as mean ± s.e.m. Asterisks denote significant difference (*P* < 0.001) of body weight between IQP G-002AS and placebo (derived from ANOVA).

*Body weight.* During the 2-week run-in phase, subjects lost an average of 1.6% of their initial body weight. At the end of the study (week 12), weight loss in the IQ arm was significantly higher compared to placebo (4.5% (3.8 kg) vs. 1.8% (1.4 kg); difference 2.4 kg (*P* < 0.001)) (Table 2). A significant difference in body weight change between IQ and placebo arms was observed as early as week 4 (*P* < 0.001) (Figure 2).

**FIGURE 3:**
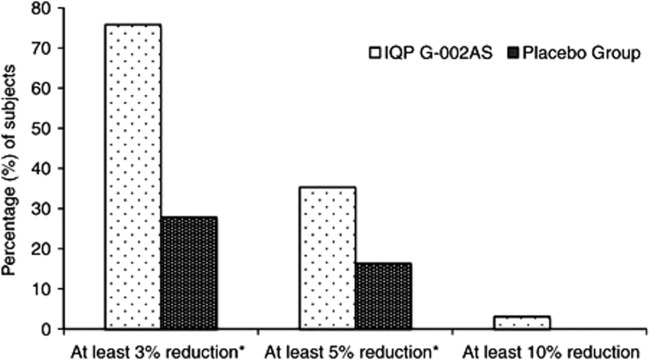
Percentage of subjects who lost at least 3, 5, and 10% of initial body weight. Asterisks denote significant difference between IQP G-002AS and placebo (derived from χ^2^).

A total of 75.8% of IQ subjects lost at least 3% of their body weight at baseline, compared with only 27.9% of placebo subjects. This difference between the two study groups was statistically significant (*P* < 0.001). There was also a significant difference in the proportion of subjects achieving at least 5% of weight loss (IQ: 35.4%; placebo 16.4%; *P* = 0.027) (Figure 3).

In the per-protocol analysis, the average body weight loss was 3.9 ± 1.8 kg in IQ-treated subjects, as compared with placebo (1.4 ± 2.6 kg). This difference between the two study groups (2.5 kg) was statistically significant (*P* < 0.001) (Table 2).

A subgroup analysis of weight loss data was carried out in 81 overweight and in 42 obese subjects. In overweight subjects, treatment with IQ resulted in more than threefold more body weight decrease compared to placebo (3.6 ± 1.8 kg vs. 0.9 ± 2.3 kg; difference 2.7 kg (*P* < 0.001)). In obese subjects, mean body weight loss in the IQ arm was about 1.8-fold compared with the placebo arm (4.3 ± 1.9 kg vs. 2.4 ± 2.9 kg; difference 1.9 kg (*P* = 0.007)).

*BMI.* At the end of the study, the IQ arm also showed significantly more reduction in mean BMI in comparison to the placebo arm (1.3 kg/m^2^ vs. 0.5 kg/m^2^ (*P* < 0.001)) (Table 3).

**Table 1:** Baseline characteristics of intention-to-treat population

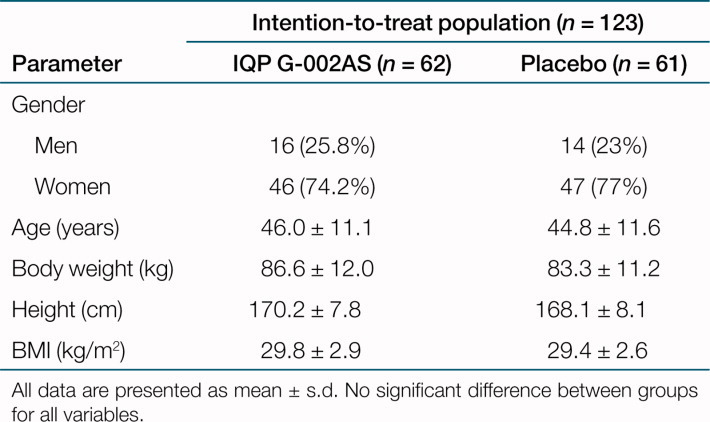

**Table 2:** Mean (± s.d.) weight loss from initial body weight, atweek 12

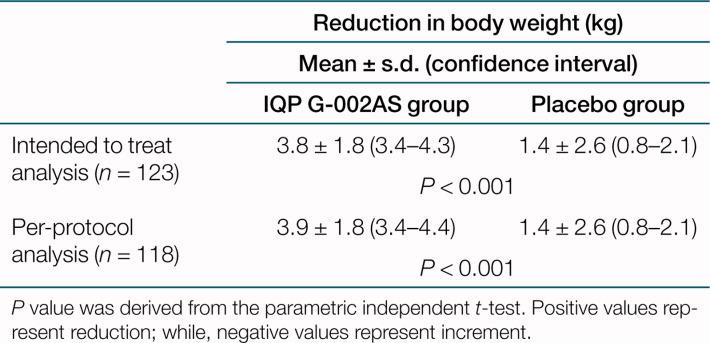

*Waist circumference.* At the end of the study, waist circumference in the IQ arm was decreased significantly more compared with the placebo arm (difference 1.7 cm; *P* < 0.001) (Table 3).

*Body fat.* The mean reduction of body fat mass was more pronounced in the IQ compared to the placebo arm. There was 1.4 kg (*P* < 0.001) or 0.8% (*P* = 0.005) difference in body fat mass between IQ and placebo arms at the end of the 12-week study (Table 3).

**Table 3:** Statistic summary of secondary efficacy parameters

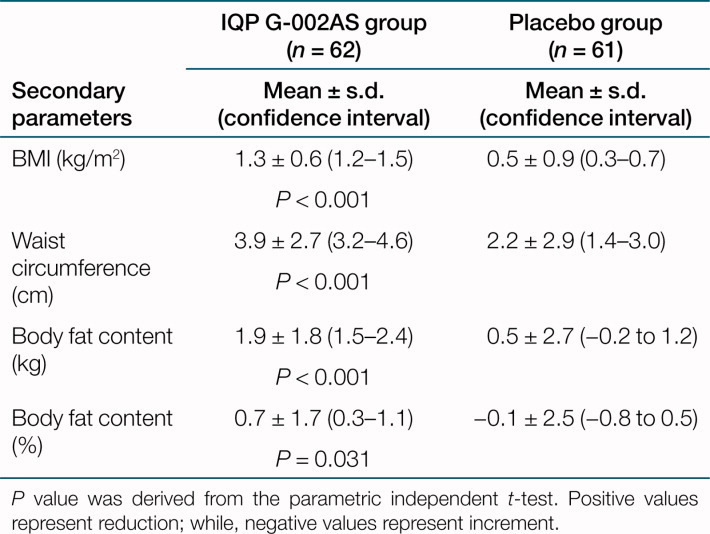

### Safety and tolerability

There were no significant changes between baseline and 12 weeks in mean heart rate and mean blood pressure. Also, no clinically relevant changes in any blood parameters were noted in the two treatment groups (Table 4).

**Table 4:** Statistic summary of safety measurements

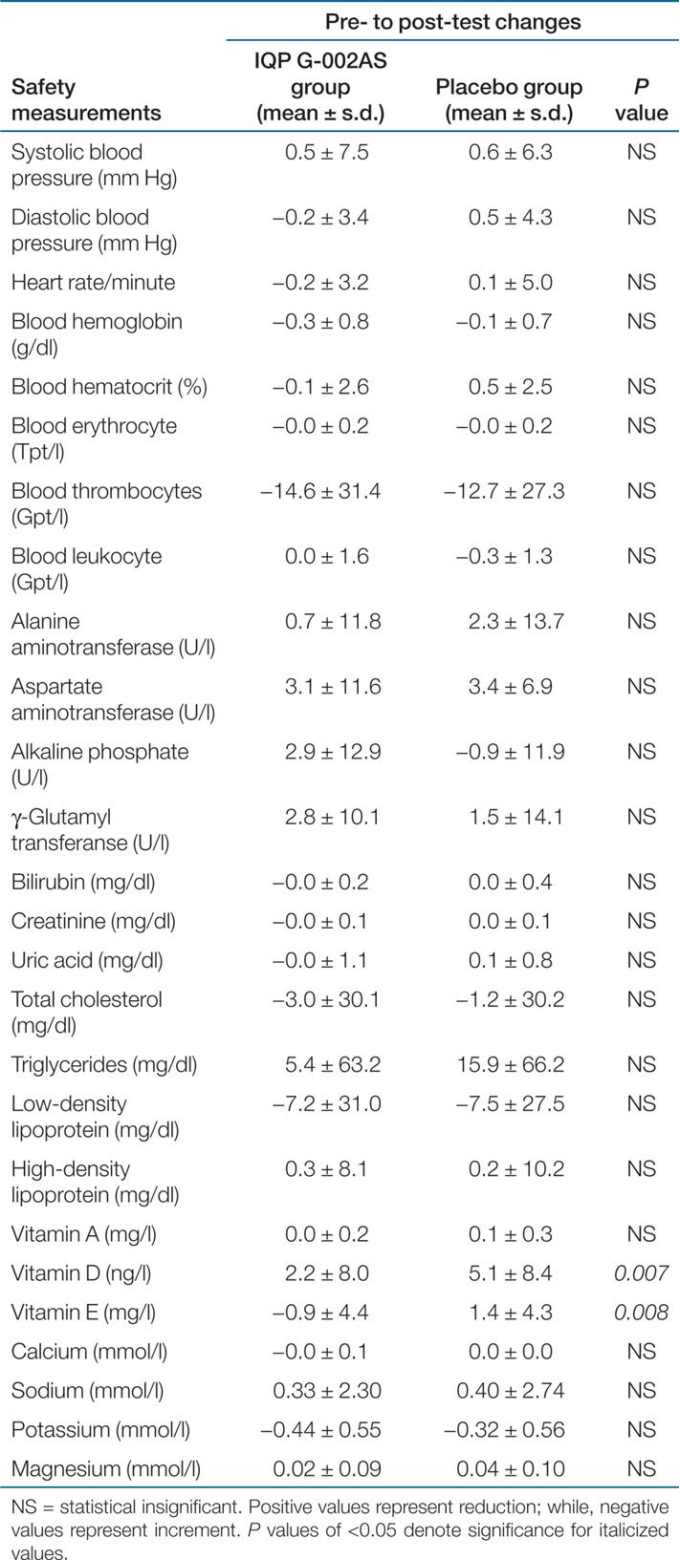

At the end of the study, 98.4% IQ subjects rated the tolerability of IQP G-002AS as “good” or “very good”, which was identical to the rating of placebo.

During the study, 26 adverse events (AEs) were documented in 24 subjects. AEs included upper respiratory tract infections and other common cold symptoms. None of the reported AE was severe and none of the reported AE was related to the intake of the study product.

## Discussion

### Weight loss effect of IQP G-002AS (primary objective)

The primary objective of this study was to demonstrate the efficacy of IQP G-002AS to induce weight loss over a period of 12 weeks in overweight and obese subjects. In this study, IQ-treated subjects lost significantly more body weight compared to placebo, with a mean difference of 2.4 kg between study arms; 35.4% of IQ-treated subjects lost at least 5% of their initial body weight. This effect may be of clinical relevance (24,25) as 5% or more of initial body weight loss reduced the odds of having metabolic syndrome, (as defined according to the criteria from the National Cholesterol Education Program's Adult Treatment Panel III) by 59% (26).

Comparisons of IQ with other weight-reducing treatments, such as chitosan (dietary fiber with similar mode of action) and orlistat (drug treatment of obesity), are difficult for methodological reasons due to lack of appropriate head-to-head studies. Jull AB *et al.* (27) reported that chitosan led to limited body weight loss (0.9 kg). The pharmacological compound orlistat at doses of 60 mg/meal (t.i.d) and 120 mg/meal (t.i.d) demonstrated mean body weight loss of 1.86 and 2.55 kg, respectively in 24 treatment weeks (28).

### BMI, body fat mass, and waist circumference

Another finding of this study is that the intake of IQP G-002AS for 12 weeks led to significant reductions of BMI, body fat content, and waist circumference. Although BMI had been used by the WHO as a standard for defining obesity, its accuracy is limited as it does not take into account physical constitution of individual factors, especially muscle and bone mass (29). Moreover, significant reduction of waist circumference in IQ-treated subjects indicates a lower central fat distribution decreasing the risk of diabetes, coronary artery disease, and hypertension. This finding is important as waist circumference is considered a more accurate marker of abdominal fat content compared to waist-to-hip ratio (25).

### Study limitations

There was no follow-up performed upon the termination of this clinical investigation. Body weight regain may occur in longer-term study, which was seen in other prior studies (12,13). Thus, future clinical investigation may aim to investigate the sustainable long-term weight loss effect of IQP G-002AS.

The second limitation of the current study is the method of measuring body fat by bio-electrical impedance analysis. The body water content may affect the accuracy of bio-electrical impedance analysis. Dual-energy X-ray absorptiometry is more accurate; however, dual-energy X-ray absorptiometry is associated with increased risk by irradiation exposure for the subjects. Hence, bio-electrical impedance analysis was used as reasonable compromise of accuracy and safety.

The effects of Litramine IQP G-002AS in improving obesity-related disease risk factors were not evaluated in the study, as the study protocol and subject inclusion were specifically designed to determine the weight loss effect of IQP G-002AS. Moreover, the study duration of 3 months is relatively short to identify the possible improvement of IQP G-002AS on the disease risk factors, accurately. Nevertheless, future intervention may be conducted to assess how long-term consumption of IQP G-002AS, combined with modest weight loss modifies the obesity-related disease risk profile.

### Subject withdrawal/compliance and safety

The administration of antiobesity drugs often leads to abdominal discomfort and is associated with liver injury and psychiatric side effects (16-,17,18), leading to high drop-out rates and low compliance (about 75%) in most of the obesity drug clinical trials; 95.2% of IQ-treated subjects completed this study indicating that IQP G-002AS is safe and well tolerated. In addition, GI side effects such as oily spotting, abdominal pain, bloating, and constipation were not reported from subjects consuming IQP G-002AS. This may be attributed to the composition of IQP G-002AS, a natural fiber complex derived from *Opuntia ficus-indica* enriched with additional soluble fiber from *Acacia spp.*, which binds to the dietary fat. The formation of IQP G-002AS natural fiber–fat complexes mitigate symptoms associated with fat malabsorption. The effectiveness of dietary fiber in diminishing the number and severity of GI side effect was demonstrated in a pancreatic lipase inhibitor study, which concluded that the mucilloid of dietary fiber prevents the GI side effect, most probably by absorbing free fat (oil) (30).

### Efficacy of IQP G-002AS under real-life conditions

Due to the very low drop-out rate in this study, the results of ITT and per-protocol populations, were consistent. The high compliance with the study protocol suggests that the regimen (IQP G-002AS plus hypocaloric diet plus moderate physical activity) may also work under in real-life conditions.

In conclusion, these results provide evidence that ingestion of the natural fiber complex Litramine IQP G-002AS represents an effective nonpharmacological intervention to induce weight loss. A follow-up study to investigate maintenance of the achieved weight loss is currently under way. Notably, this beneficial effect was achieved without side effects.
